# Retraction Note to: *Linc00210* drives Wnt/β-catenin signaling activation and liver tumor progression through CTNNBIP1-dependent manner

**DOI:** 10.1186/s12943-022-01633-6

**Published:** 2022-08-10

**Authors:** Xiaomin Fu, Xiaoyan Zhu, Fujun Qin, Yong Zhang, Jizhen Lin, Yuechao Ding, Zihe Yang, Yiman Shang, Li Wang, Qinxian Zhang, Quanli Gao

**Affiliations:** 1 Department of Cancer Biology Immunotherapy, The Affiliated Cancer Hospital of Zhengzhou University and Henan Cancer Hospital, 127 Dongming Road, Zhengzhou, 450003 Henan China; 2 Department of Histology and Embryology, College of Basic Medicine, Zhengzhou University, 100 Kexue Road, Zhengzhou, 450052 Henan China; 3 Department of Pathology, School of Medicine, University of Virginia, Charlottesville, VA 22908 USA; 4 Department of Otolaryngology, Medical School, University of Minnesota, Twin Cities Campus, Minneapolis, MN 55414 USA; 5 Department of Hepatopancreatobiliary Surgery, The Affiliated Cancer Hospital of Zhengzhou University and Henan Cancer Hospital, Zhengzhou, 450003 Henan China; 6 Department of Nuclear Medicine, The Affiliated Beijing Anzhen Hospital of Capital Medical University, Capital Medical University, Beijing, 100029 China

**Retraction Note to: Mol Cancer 17, 73 (2018)**

**https://doi.org/10.1186/s12943-018-0783-3**

The Editor-in-Chief has retracted this article at the Authors' request. After publication, concerns were raised regarding suspected image overlap with a previously published article [1]. Specifically, Fig. 6H Sample#1 CTNNBIP1#2 in this article and Fig. 2F HCC sample shC8#2 in [1] appear to originate from the same sample. The Authors have stated that they are unable to share parts of the original dataset and the ethics approval documentation.

The Editor-in-Chief therefore no longer has confidence in the presented data.

All authors agree to this retraction.
